# Management of Statin Intolerant Patients in the Era of Novel Lipid Lowering Therapies: A Critical Approach in Clinical Practice

**DOI:** 10.3390/jcm12062444

**Published:** 2023-03-22

**Authors:** Giosiana Bosco, Francesco Di Giacomo Barbagallo, Salvatore Spampinato, Lorena Lanzafame, Antonino Di Pino, Salvatore Piro, Francesco Purrello, Roberto Scicali

**Affiliations:** Department of Clinical and Experimental Medicine, University of Catania, Internal Medicine, Garibaldi Hospital, Via Palermo 636, 95122 Catania, Italy

**Keywords:** statin intolerance, non-statin lipid lowering therapy, LDL-C target, therapeutic adherence, cardiovascular risk reduction

## Abstract

Statins are the cornerstone of lipid-lowering therapies effective for cardiovascular risk reduction. Although they are generally well tolerated, statin intolerance (SI) is frequent in clinical practice, and it is usually related to the onset of muscle symptoms, which are defined under the acronym SAMS (Statin-Associated Muscle Side Effects). These side effects are responsible for statin treatment discontinuation that results in increased cardiovascular risk. The National Lipid Association (NLA) has recently provided an updated definition of statin intolerance, and a distinction between complete and partial statin intolerance has been reported. The evaluation of symptom severity and the presence of muscle damage biomarker alterations make it essential to adopt a patient-centered approach aimed at obtaining a personalized therapeutic strategy. Firstly, it could be useful to administer a different statin, reduce the dosage or adopt an alternate dosage regimen. However, some patients are unable to tolerate any statin at every dosage, or despite taking statins at the maximum tolerated dose, they fail to achieve the recommended LDL-C target, and thus it is necessary to introduce a non-statin hypolipidemic treatment. Ezetimibe, proprotein-convertase subtilisin/kexin type 9 (PCSK9) inhibitors such as monoclonal antibodies (alirocumab and evolocumab) or RNA messenger silencing (inclisiran), bempedoic acid or nutraceuticals are non-statin lipid-lowering therapies that could be used as an alternative or in addition to statins to achieve an early and sustained LDL-C reduction in clinical practice. In this review, we evaluated SI management focusing on non-statin lipid lowering therapies and their implications in lipid lowering approaches in clinical practice.

## 1. Introduction

Atherosclerotic cardiovascular disease (ASCVD) is the worldwide leading cause of morbidity and mortality, and an increased low-density lipoprotein cholesterol (LDL-C) plasma level has been considered the causal factor of atherosclerosis progression [1]. However, mendelian randomization studies as well as randomized controlled clinical trials and systematic meta-analyses demonstrated that a low LDL-C plasma level was associated with a low cardiovascular risk that was more pronounced if an early and durable LDL-C reduction was observed [2].

Statin therapy is considered the first LDL-C lowering strategy able to reduce the cholesterol synthesis by inhibiting 3-hydroxy-3-methylglutaryl CoA (HMGCoA) reductase; thus, liver cholesterol depletion leads to low-density lipoprotein receptor (LDLR) upregulation on the hepatocyte surface and to an increased uptake of circulating LDL-C [3]. According to their solubility, statins are classified as lipophilic (simvastatin, atorvastatin, lovastatin, fluvastatin and pitavastatin) or hydrophilic (rosuvastatin and pravastatin); moreover, based on the lipid-lowering efficacy, we recognize low-intensity (<30% LDL-C reduction, fluvastatin 20–40 mg, lovastatin 20 mg, pravastatin 20 mg, simvastatin 10 mg), moderate-intensity (30–49% LDL-C reduction, fluvastatin XL 80 mg, lovastatin 40 mg, pravastatin 40 mg, simvastatin 20–40 mg, atorvastatin 10–20 mg, rosuvastatin 5–10 mg) or high-intensity (≥50% LDL-C reduction, atorvastatin 40–80 mg, rosuvastatin 20–40 mg) statin therapies [4]. Thus, in the era of personalized medicine the choice of statin dosage depends on cardiovascular risk level as well as the baseline LDL-C level to achieve the recommended LDL-C target [5].

Although statin therapy is generally well tolerated, side effects as well as treatment discontinuation have been reported in clinical practice [6]; moreover, it was shown that an impaired statin therapy adherence was associated with LDL-C off target and ASCVD risk increase [7].

Statin Associated Muscle Symptoms (SAMS) are the most frequent side effects causing statin discontinuation. SAMS have several causes, firstly a negative drucebo effect where damage expectation results from perceived side effects while these symptoms are probably not related to statin mechanism. It is essential to evaluate and properly identify any statin intolerance (SI, partial or complete) to manage statin intake better and choose the appropriate therapeutic strategy for LDL-C reduction. In this context, it was previously showed that non-statin lipid lowering therapies such as ezetimibe or proprotein convertase subtilisin/kexin-type 9 inhibitors (PCSK9-i) were able to reduce LDL-C and ASCVD risk [8,9]; moreover, PCSK9 RNA silencing (siRNA) as well as ATP citrate lyase inhibitor (ACLY) have recently shown a significant LDL-C decrease [10,11]. In this review, we evaluated SI management focusing on non-statin lipid lowering therapies and their implications in lipid lowering approaches in clinical practice.

## 2. Methodology for Literature Searching

This review was performed based on the English language articles on statin intolerance obtained from the Pubmed, Springer, Elsevier, Multidisciplinary Digital Publishing Institute (MDPI) and Web of Science databases. Several keywords have been used such as “statin intolerance AND muscle symptoms”, “discontinuation of statin treatment AND cardiovascular risk”, “therapeutic adherence AND LDL-C target” and “statin intolerance AND non-statin lipid lowering therapy”. Selected articles included randomized controlled trials (RCT), meta-analysis, systematic reviews and clinical guidelines. While articles addressing statin-associated muscle symptoms were examined, in vitro studies regarding the biochemistry of statin-associated muscle symptoms have not been considered. Articles describing possible mechanisms of statin intolerance and the prevalence of negative drucebo effects were also evaluated. Clinical studies focusing on the non-adherence and/or discontinuation of statin therapy and its impact on cardiovascular and cerebrovascular events as well as all-cause mortality have been considered. Finally, trials showing the benefits of non-statin lipid lowering therapies have also been evaluated.

## 3. Statin Intolerance

### 3.1. Definition and Clinical Manifestations

Over the years, several SI definitions have been proposed to better identify this condition in clinical practice; recently, the National Lipid Association (NLA) provided an updated and accurate SI definition to ameliorate its identification and management [12]. According to the NLA scientific statement, SI is defined as one or more statin related adverse effect that resolves or improves after statin dosage reduction or discontinuation and can be classified as complete inability to tolerate any dose of statins or partial intolerance that is defined as the inability to tolerate the needed dosage to achieve LDL-C target. SI is considered if at least two statins have been assumed where one of them is at the lowest approved daily dosage [12]. Statin associated side effects (SASE) include a broad spectrum of clinical manifestations such as myalgia, myopathy, rhabdomyolysis, statin induced autoimmune myopathy, newly diagnosed diabetes, liver injury, renal injury, hemorrhagic stroke, cognitive impairment, cataract, cancer and tendon injury (Table 1).

It is estimated that in clinical practice almost 10% of patients discontinue statins due to SASE or the fear of its development; among SASE, the most frequently observed disorder as well as the main reason for statin discontinuation is the onset of statin associated muscle symptoms (SAMS) [14]. SAMS include myalgia that is defined as a feeling of weakness or symmetrical soreness in proximal muscles without creatine kinase (CK) increase, myopathy that is considered an unexplained muscle pain or weakness associated with an increase in creatine kinase (CK) concentration > 10 times the upper limit of normal (ULN) and finally rhabdomyolysis that is described as a severe form of myopathy with markedly elevated CK levels (often >40 times the ULN) requiring hospitalization and associated with acute renal failure; however, these clinical conditions disappear after statin therapy discontinuation. In a low percentage of SAMS, it is reported that the statin-associated autoimmune myopathy (SAAM) is characterized by the presence of proximal muscle weakness and significantly elevated CPK levels (often >10 times the ULN) that do not improve with statin cessation; HMGCoA-reductase antibody testing is used for confirming the diagnosis. SAAM could have an early onset, but usually it appears after several years of statin therapy with an incidence of 2–3 per 100,000 patients treated with statins.

A recent meta-analysis from randomized clinical trials showed that the difference of myalgia incidence due to statins or placebo was <1% [15]; this finding suggests that the onset of muscle disorders during statin therapy may be at least attributable to a negative drucebo effect, where damage expectation results in perceived side effects that may not be related to the drug [16]. The available data on the statin related negative drucebo effect suggest adopting a statin intolerance prevention system program useful to reduce the probability of an erroneous SAMS diagnosis. To improve long term statin adherence, patients should be informed about the LDL-C lowering efficacy and the cardiovascular benefit of statin therapy; moreover, patients should be advised about the real likelihood of statin related adverse events. Furthermore, in patients with a family history of statin intolerance or a high risk of statin intolerance (for example in cases of hepatic/renal impairment, polypharmacotherapy, etc.), a suitable statin regimen should be considered to avoid side effect occurrence [7]. It is needed for clinicians to monitor patients after statin prescription. In this context, a frequent follow-up should be scheduled in order to evaluate the efficacy of treatment, the adherence to therapy and the onset of adverse effects. Patient participation in decision-making with the continuous education is critical to overcome the negative drucebo effect and to avoid a suboptimal lipid-lowering therapy.

As concerns the pediatric population, it was shown that the risk of statin treatment associated adverse event was lower in children than in adults. Two possible explanations may be considered. Firstly, in children a low statin dose is usually considered as starting therapy; moreover, the pediatric population has generally less frequent comorbidities that could increase drug to drug interactions and SAMS risk. Of note, the prevalence of adverse events was similar between children receiving statins or placebo. If adverse events occur, they are nonserious and reversible events and principally include the liver toxicity, SAMS, teratogenic effects and drug-to-drug interaction. Despite the reported safety and efficacy, many children with lipid disorders are not on statin therapy; in this context, it is extremely important to early identify and manage children with lipid abnormalities in order to prevent their future cardiovascular risk [17].

**Figure 1:**
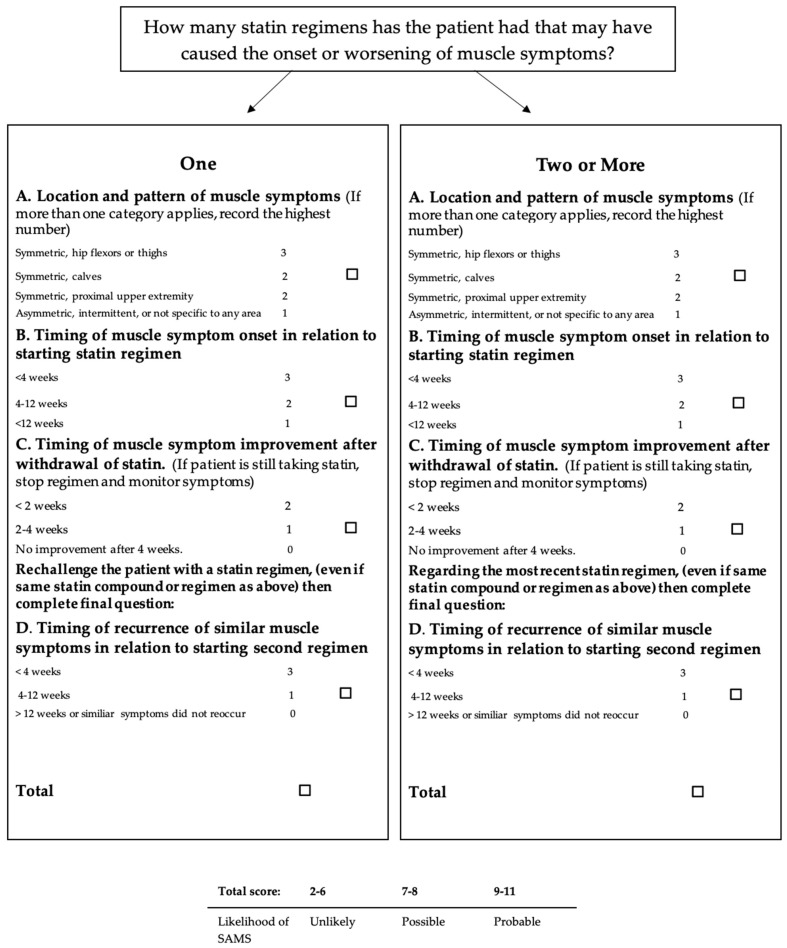
Statin-Associated Muscle Symptoms Clinical Index (SAMS-CI) adapted from Rosenson et al. 2017 [19].

However, the prevalence of statin intolerance is higher in clinical practice than in RCTs, and it is probably due to the use of non-unique terminology of SAMS description or the lack of standardized measurements for SAMS detection. Thus, in order to determine how likely muscle symptoms are attributable to statins, the NLA Statin Muscle Safety Task Force performed the Statin-Associated Clinical Index of Muscle Symptoms (SAMS-CI) [18]. The SAMS-CI includes four separate items: the first regards the location and patterns of muscle symptoms, and the others focus on the timing of symptoms relative to starting, stopping (dechallenge) and rechallenging with statins (Figure 1).

In this context, in order to optimize the lipid lowering therapy in subjects with muscle symptoms, SAMS-CI could be useful for the detection of statin-related muscle disorders, and thus a personalized lipid lowering therapy approach could be adopted [19]. Several endogenous and exogenous factors have been identified to promote the onset of SAMS (Table 2).

The endogenous factors include gender, age, ethnicity and genetic factors. Gheorghe et al. showed that women are more likely to discontinue statin treatment compared to men (10.9% vs. 6.1%) as well as to take a lower statin dose than recommended (3.6% vs. 2%); these findings could be explained by increasing fear of adverse reactions such as muscle symptoms and hepatic cytolysis. In this context, it is needed to focus on the differences of gender related statin metabolism. Women have a lower muscle mass than men, and so they might be more vulnerable to SAMS; in contrast, they have a higher percentage of fat tissue compared to men with increasing the lipophilic statin distribution volume. Finally, the CYP3A4 enzyme expression is twice higher in women than in men, and thus the SAMS risk could be increased in female subjects by enhancing CYP450 isoform interactions. Taking into consideration these findings, the gender-related statin metabolism might explain why women are more exposed to the risk of statin discontinuation and thus to an increased risk of future cardiovascular events. In this context, it is needed to optimize statin treatment in women to improve their compliance and to achieve the recommended LDL-C targets [21].

Moreover, the exogenous factors include alcohol or drug consumption, extreme exercise or concomitant therapies with potential drug-to-drug interactions such as protease inhibitors, azoles, macrolides or immunosuppressants [22,23,24].

While atorvastatin, lovastatin and simvastatin are metabolized primarily by the isoenzyme P3A4 of cytochrome P450 (CYP3A4), the isoenzyme P2C9 (CYP2C9) is responsible for metabolism of fluvastatin, pitavastatin and rosuvastatin. Moreover, pravastatin is the only statin that is not metabolized by the CYP isoenzyme family.

Atorvastatin, simvastatin and lovastatin are mainly involved in drug-to-drug interactions; in this context, cytochrome P3A4 inhibitors such as azole antifungals, immunosuppressive agents and human immune-deficiency virus (HIV) protease inhibitors can greatly increase statin plasma concentration with increasing the risk of statin intolerance. These are drugs widely used in clinical practice, and strong epidemiologic evidence has demonstrated that in autoimmune disorders or in case of HIV infection there is a premature, rapidly processing atherosclerosis development, followed by cardiovascular (CV) complications [25]. Moreover, bleeding or prolonged prothrombin time has been reported in patients taking statins metabolized by CYP3A4 concomitantly treated with warfarin [26]. In this context, the discontinuation of statin treatment due to drug-to-drug interactions might be associated with an increased risk of CV diseases and all-cause mortality.

The assessment and the correction of modifiable risk factors could reduce the adverse effects of statin therapy. 

As concerns glucose homeostasis, the absolute risk of statin-related newly diagnosed diabetes mellitus is about 0.2% per year, although this percentage depends on the population risk, and it could be greater in patients with a family history of diabetes mellitus as well as subjects with pre-diabetes or insulin resistance [15].

As regards liver injury, while severe liver toxicity is rare (0.001%), an increase of transaminases > 3 times the ULN is common, but it is usually transient and not associated with hepatopathy; however, this alteration is usually managed by stopping statins and rechecking transaminases after 4–6 weeks [26].

Finally, there is no evidence of a causal relationship between statin use and risks of hemorrhagic stroke, cognitive impairment, cataract or cancer [27].

### 3.2. Statin Intolerance and Cardiovascular Events

While the lipid-lowering effects of statins typically occur within 4 weeks of initiating therapy, the treatment-related cardiovascular benefits are usually observed after at least 2–5 years of continuous therapy [28].

Depending on statin doses, it was shown that LDL-C as well as triglycerides (TG) were reduced by 20–60% and 10–30%, respectively [29]; moreover, a meta-analysis of randomized clinical trials showed that an LDL-C decrease of 1 mmol/L (39 mg/dL) was associated with 10%, 23% and 17% reductions of all cause-mortality, coronary events and ischemic stroke, respectively [30]. On the other hand, Sandoval et al. showed that a poor adherence or discontinuation of statin therapy were linked to increased cardiovascular disease, cerebrovascular events or mortality [28].

Finally, Serban et al. found that the cumulative incidence of recurrent myocardial infarction was higher among patients with statin intolerance compared to high adherence statin subjects [31].

## 4. Management of Statin Intolerance

The evaluation of patients with symptoms suggestive of statin intolerance includes a complete medical history as well as an accurate physical examination and the assessment of muscle damage biomarkers. Thus, the physician has collected information about the assumed statin type and dosage, and thus the SAMS-CI score could be performed to evaluate the probability of statin related muscle symptoms.

It was shown that the majority of SAMS occurred in the first 12 weeks of statin treatment [32].

**Figure 2:**
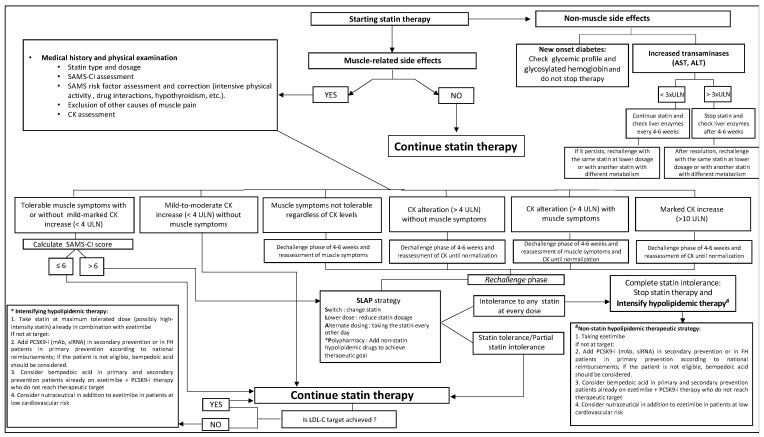
Algorithm for the management of patients with statin intolerance. Abbreviations: ALT = alanine transaminase, AST = aspartate aminotransferase, CK = creatine kinase, CV = cardiovascular, FH = familial hypercholesterolemia, LDL-C = low-density lipoprotein cholesterol, mAb = monoclonal antibodies, PCSK9-i = proprotein convertase subtilisin/kexin type 9 inhibitor, SAMS= Statin-associated muscle symptom, SAMS-CI = statin-associated muscle symptoms clinical-index, siRNA = small-interfering RNA, SLAP = switch–lower dose–alternate dose–polypharmacy, ULN = upper limits of normal.

Estimating the severity of symptoms and possible muscle damage biomarker alteration is needed to adopt a patient-centered approach to effectively manage statin intolerance [33] (Figure 2).

If a mild or moderate increase of CK (<4 ULN) without symptoms is present, it is safe to continue statin therapy. However, when the patient reports tolerable symptoms, with or without mild to moderate CK elevation (<4× ULN), it is recommended to use the SAMS-CI clinical score to assess the causality between statin therapy and muscle adverse effects. If the SAMS-CI score is <6 it is unlikely that statin therapy is responsible for muscle symptoms that may be related to the nocebo effect; thus, patients should be motivated to continue statin therapy. Conversely, if the SAMS-CI score is ≥6, SI probability increases; in this context, lipid lowering treatment management is summarized in the acronym SLAP (Switch, Lower dose, Alternate dosing, Polypharmacy) [34]:

- Switch

Statin intolerance may be related to the specific type of assumed statin; thus, it could be useful to change lipophilic (simvastatin, atorvastatin, fluvastatin, lovastatin) with hydrophilic (pravastatin or rosuvastatin) statins or vice versa.

- Lower dose

It has been shown that SAMS were dependent on statin dose, and thus a low dose drug administration could reduce muscle symptoms. Moreover, it was shown that subjects on low dose statins exhibited a reduced ASCVD occurrence compared to non-statin users [34]. If a low dose statin is tolerated, it is possible to gradually increase the dose over time.

- Alternate dosage regimen

An additional strategy to reduce the occurrence of side effects is to take statins on alternate days; in this context, several RCTs demonstrated that no significant differences of LDL-C and TG reductions were found between daily and alternate day statin use [35].

- Polypharmacy

In partial or complete statin intolerant patients, a polypharmacy strategy to achieve the LDL-C target is needed. However, in the DA VINCI Study, it was shown that only 55% of patients achieved their LDL-C goal based on the 2016 ESC/EAS dyslipidemia guidelines, and this percentage was lower according to the 2019 ESC/EAS dyslipidemia guidelines; moreover, the prevalence of primary or secondary prevention patients at very high-risk who reached the recommended 2016 ESC/EAS dyslipidemia guideline LDL-C target were 22% and 45% (17% and 22% for 2019 ESC/EAS guidelines), respectively. Furthermore, a high-intensity statin monotherapy was used in 20% and 38% of primary and secondary prevention patients at very high-risk, respectively, and the use of moderate-to-high-intensity statins in combination with ezetimibe or with PCSK9 inhibitors was low (9% and 1%, respectively). Overall, combination therapy was associated with a higher rate of LDL-C target achievement than statin monotherapy; in particular, 37% and 57% of patients receiving the combination therapy of statin plus ezetimibe or PCSK9 inhibitor achieved LDL-C goal, respectively [36]. Moreover, in the SANTORINI Study it was found that 20% of patients at high or very high risk achieved the LDL-C goals, and only 27% of patients were on combination therapy, including statin plus ezetimibe (17%) or PCSK9 inhibitors (4.1%) [37]. In this context, the addition of non-statin lipid-lowering therapy (ezetimibe, PCSK9 inhibitors, bempedoic acid, nutraceuticals) could be useful to reach the recommended LDL-C target.

If the patient documents not tolerable muscle symptoms, statin therapy should be interrupted for 4–6 weeks independently of muscle damage biomarker alteration (dechallenge phase). After re-evaluation of muscle symptoms, statin therapy could be resumed (rechallenge phase), and the SLAP algorithm could be considered. If a muscle damage biomarker alteration is reported (CK > 4× ULN), statin should be suspended until its normalization (dechallenge phase), and then it could be resumed (rechallenge phase) with considering SLAP strategy. In this context, the PROSISA study showed that in a cohort of dyslipidemic patients with reported statin intolerance, the majority did not exhibit SAMS after the SLAP algorithm [16].

If a complete statin intolerance is reported despite the SLAP strategy or partial statin intolerant patients do not achieve the recommended LDL-C targets, a non-statin lipid lowering therapeutic approach should be considered.

## 5. Non-Statin Lipid Lowering Treatments

**Figure 3:**
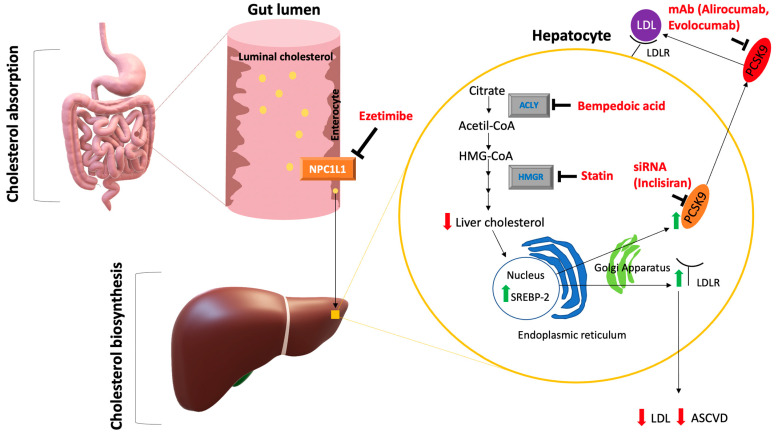
Mechanism of action of lipid-lowering drugs. Abbreviations: ACLY = ATP citrate lyase, ASCVD = atherosclerotic cardiovascular disease, HMGR = HMG-CoA reductase, LDL = low density lipoprotein, LDLR = low density lipoprotein receptor, mAb = monoclonal antibodies, NPC1L1 = Niemann-Pick C1-Like 1, PCSK9 = proprotein convertase subtilisin/kexin type 9, siRNA = small interfering RNA, SREBP-2 = sterol regulatory element binding protein 2.

Since LDL-C is the causal factor of the atherosclerotic process, non-statin lipid lowering therapies able to indirectly increase the liver LDLR expression and thus effectively reduce LDL-C should be considered in statin intolerant patients (Figure 3).

### 5.1. Ezetimibe

Ezetimibe is a selective inhibitor of the Niemann–Pick transporter C 1 like 1 (NPC1L1) on enterocytes, and thus it reduces the intestinal cholesterol absorption [3]. It was shown that after 4–6 weeks ezetimibe monotherapy causes an LDL-C reduction by 15–22% that slowly diminishes in combination with statins [38]. Moreover, ezetimibe has low pharmacokinetic interactions with drugs metabolized by cytochrome P450 with little probability of muscle adverse effects.

The IMPROVE-IT study showed that in patients with a history of acute coronary syndrome the addition of ezetimibe to simvastatin lowered LDL-C levels by 24% compared to simvastatin alone; moreover, a reduction of cardiovascular events was observed in patients who assumed ezetimibe [9]. Furthermore, in subjects at high risk of cardiovascular events such as familial hypercholesterolemia, ezetimibe effectively reduced the progression of vascular injury [39].

Taking into consideration these findings, ezetimibe should be started as soon as possible in high or very high cardiovascular risk patients with partial or complete statin intolerance, even during the statin withdrawal phase [40].

### 5.2. PCSK9 Inhibitors

Proprotein subtilisin/kexin convertase type 9 (PCSK9) is a regulatory protein that binds to LDLR on the hepatocyte thus promoting its degradation. Two lipid lowering strategies against PCSK9 are available in clinical practice: the monoclonal antibodies (mAbs) alirocumab and evolocumab and the small interfering RNA (siRNA) inclisiran. The mAb mechanism of action is to bind and inhibit circulating PCSK9; thus, these drugs prevent LDLR degradation and enhance its expression on the hepatocyte surface. It was previously reported that every 2 weeks subcutaneous PCSK9-mAbs administrations effectively reduced LDL-C by 50–60%; thus, based on their efficacy these drugs are needed in subjects with extreme LDL-C levels such as FH [41]. Moreover, a significant 20–25% lipoprotein(a) [Lp(a)] reduction was observed after PCSK9-mAbs treatment; currently, this novel lipid lowering therapy represents the only available non-invasive lipid-lowering strategy to reduce Lp(a) plasma levels. Furthermore, The ODYSSEY-ALTERNATIVE and GAUSS-3 studies reported that in statin intolerant patients alirocumab and evolocumab significantly lowered LDL-C levels compared to ezetimibe and these PCSK9-mAbs exhibited acceptable safety profiles [42,43]. Finally, The FOURIER and ODYSSEY-Outcomes studies demonstrated that in subjects with a history of ASCVD evolocumab and alirocumab effectively reduced the risk of cardiovascular events [44,45]. Thus, in FH or very high cardiovascular risk subjects, the early administration of PCSK9-mAbs in addition to ezetimibe and maximally tolerated statin dose should be considered to achieve the recommended LDL-C targets. Inclisiran is a small interfering RNA (siRNA) that binds the PCSK9 RNA messenger and selectively inhibits PCSK9 synthesis in the liver. This drug was approved by the FDA in December 2021 for adults with familial hypercholesterolemia or atherosclerotic cardiovascular disease who required further LDL-C reduction despite maximally tolerated statin therapy [46]. A combined analysis of the ORION-9, ORION-10 and ORION-11 studies showed that in FH subjects or in subjects with a history of cardiovascular events twice year inclisiran administrations significantly reduced LDL-C levels by 50% with a good safety profile [10]. Recently, it was shown that in a 2 year prespecified analysis inclisiran effectively reduced the composite endpoint of major adverse cardiovascular events while longer studies are needed to evaluate its efficacy for non-fatal cardiovascular event reduction [47]. Taking into consideration its pharmacokinetic properties, inclisiran could exhibit the highest therapeutic adherence and persistence in clinical practice, and thus it could be a useful lipid lowering therapy in statin intolerant patients [48,49].

### 5.3. Bempedoic Acid

Bempedoic acid inhibits the adenosine triphosphate-citrate lyase (ACLY) that is involved in cholesterol biosynthesis by catalyzing the conversion of citric acid in oxaloacetate and acetyl-CoA [46]. Thus, bempedoic acid increases the LDLR expression on the hepatocyte surface, and thus a reduction of LDL-C levels is observed. It is a pro-drug converted into its active form (bempedoic-acid CoA) by very long-chain acyl-CoA synthetase 1 (ASCVL1) that is only localized in the liver, and thus the likelihood of developing muscle side effects is reduced by bempedoic acid treatment. In the CLEAR Serenity study, it was shown that bempedoic acid therapy lowered LDL-C by 21.4% compared to placebo [50]; moreover, Ballantyne et al. found that a 38% LDL-C reduction was observed after addition of a bempedoic acid plus ezetimibe fixed dose combination in statin intolerant subjects at high cardiovascular risk [51]. Furthermore, in two phase 2 randomized clinical trials Rubino et al. demonstrated that a triple therapy with atorvastatin 20 mg, ezetimibe 10 mg and bempedoic acid 180 mg significantly lowered LDL-C levels by 60.5% compared to placebo [52] while the addition of bempedoic acid to PCSK9i promoted a 30% LDL-C reduction compared to placebo, which could be useful in statin intolerant subjects with an elevated baseline LDL-C such as FH [53].

The ongoing CLEAR Outcomes study will be the first trial that will assess the effect of bempedoic acid treatment on the cardiovascular outcomes in patients with statin intolerance.

### 5.4. Nutraceuticals

Nutraceuticals consist of nutrients or bioactive compounds derived from plants or microbes and characterized by a lipid lowering effect [54]. According to their mechanism of action, nutraceuticals can be distinguished into the inhibitors of intestinal cholesterol absorption (plant sterols and stanols, soluble fibers, oat fibers, psyllium, glucomannan and probiotics), inhibitors of hepatic cholesterol synthesis (red yeast rice, garlic, bergamot and artichoke) or LDL-C clearance enhancers (berberine, green tea extracts and soy); of these, red yeast rice, polycosanols and berberine are the most used in clinical practice. A recent meta-analysis of 12 double-blind placebo-controlled randomized trials involving 1050 subjects suggested that nutraceutical supplementation had a beneficial effect on body mass index as well as serum LDL-C, TG, HDL-C, high sensitivity C-reactive protein and fasting plasma glucose, and it showed good safety and tolerability profiles [55]. However, no consistent data exist regarding the effect of nutraceuticals on cardiovascular outcomes. Taking into consideration these findings, nutraceuticals could be considered in statin intolerant patients at low cardiovascular risk [56].

### 5.5. Other Therapeutic Strategies for LDL-C Reduction

Novel genetic therapies are ongoing in the treatment of dyslipidemia [57]. Gene-based treatments offer great potential and possibilities to regulate key points in lipid metabolism with high specificity and efficacy. DNA-targeted therapies such as adeno-associated virus or CRISPR–Cas9 modification could be used to edit the genetic status with improving the related phenotype. Moreover, RNA-targeted therapies such as antisense oligonucleotides (ASOs) and siRNA can regulate genes by directly targeting the nucleic acids that encode the proteins and by interfering with the translation of mRNA into protein. ASOs-based approaches include mipomersen, an antisense oligonucleotide targeting ApoB100 actually available only in the USA for homozygous familial hypercholesterolaemia and vupanorsen, an antisense oligonucleotide targeting angiopoietin-like protein (ANGPLT3). In addition to inclisiran, siRNA therapies also include ARO-ANG3, a small interfering RNA that inhibits the transcription of ANGPTL3 mRNA in liver cells [58]. This promising novel genetic therapies might be considered especially in patients with high CV risk and statin intolerance beyond the recommended LDL-C target. Metformin, Glucagon-like peptide 1 (GLP-1) agonists, glucose-dependent insulinotropic polypeptide (GIP) agonists and dual GLP-1/GIP agonists used for the treatment of diabetes mellitus and obesity might be considered as an alternative strategy in diabetic and dyslipidemic overweight patients with statin intolerance to improve the lipid profile by reducing weight. Moreover, in dyslipidemic obese patients, novel therapeutic strategies targeting the endocannabinoid system might also be considered [59].

## 6. Conclusions

Statins are the cornerstone of LDL-C lowering therapies and play a key role in cardiovascular disease prevention. However, statin intolerance, which is mainly manifested with the onset of SAMS, is responsible for treatment discontinuation. Since it is estimated that complete statin intolerance is quite rare and that most of the symptoms are attributable to a negative drucebo effect, multiple strategies should be considered before stopping statin treatment. Several non-statin lipid-lowering therapies have shown efficacy and safety on LDL-C reduction, and these treatments, alone or in combination, could be useful strategies for LDL-C lowering in clinical practice. A personalized lipid lowering approach should be considered to obtain a sustained therapeutic adherence that is needed to achieve the recommended LDL-C target.

## Figures and Tables

**Table 1:** Side effects associated with statin intake and their frequency adapted from Grundy et al. (2019) [13].

Statin-Associated Side Effects (SASE)	Frequency
Statin-Associated Muscle Symptom (SAMS)	
- Myalgia: feeling of weakness or symmetrical soreness in proximal muscles (normal CK)	Infrequent (1–5%) in RCTs; frequent (5–10%) in observational studies and in clinical practice
- Myopathy: unexplained muscle pain or weakness or both	Rare
- Rhabdomyolysis: Severe form of myopathy requiring hospitalization and associated with renal failure and myoglobinuria (CK > 40 ULN)	Rare
- Statin-associated autoimmune myopathy: myopathy characterized by the presence of anti-HMGCoAR antibodies and incomplete resolution of pain symptoms	Rare
New onset diabetes mellitus	It depends on population. Frequency increases in the presence of diabetes mellitus risk factors, such as BMI ≥ 30, fasting blood glucose ≥ 100, metabolic syndrome, HbA1c ≥ 6%
Hepatic injury	
- Increase of transaminases 3xULN	Infrequent
- Liver failure	Rare
Central nervous system	
- Cognitive deterioration	Rare
Cancer	No clear association
Kidney disease	No clear association
Cataract	No clear association
Tendon rupture	No clear association
Haemorrhagic stroke	No clear association
Interstitial lung disease	No clear association
Hypogonadism	No clear association

Abbreviations: BMI = body mass index, CK = creatine kinase, HbA1c = glycosylated hemoglobin, HMGCoAR = 3-Hydroxy-3-Methylglutaryl-CoA Reductase, RCTs = randomized controlled trials, ULN = upper limits of normal.

**Table 2:** Endogenous and exogenous risk factors predisposing SAMS adopted from Gulizia et al. 2017 [20].

Endogenous Risk Factors	Exogenous Risk Factor
Elderly	Intensive physical activity
Female sex	High intensity statin therapy
Asian ethnicity	Alcohol abuse
Positive history of muscle and/or joint pain	Drug abuse
Inflammatory or metabolic neuromuscular disease - Spinobulbar muscular atrophy - Alpha 1 4 glucosidase deficiency - Carnitine palmitoyl transferase II deficiency - Dermatomyositis - Myotonic dystrophy types I and II - Malignant hyperthermia - Mcardle’s disease - Myasthenia gravis - Recurrent myoglobinuria - Mitochondrial myopathy - Necrotizing myopathy - Inclusion body myositis - Peripheral neuropathy - Polymyositis - Amyotrophic lateral sclerosis	Drug interactions - Nicotinic acid - Amiodarone - Azoles - Antipsychotic drugs - Immunosuppressive drugs (cyclosporine) - Fibrates (gemfibrozil) - Protease inhibitors - Macrolides - Nefazodone - Verapamil - Warfarin
Positive history of increased CK (especially if CK > 10 ULN)	Grapefruit or blueberry juice consumption (>1 L/day)
Positive family history of myopathy	Unregulated supplements (e.g., red yeast rice, pleurotus mushrooms, etc.).
Induced myopathy by statins or other hypolipidemic drugs	Surgical procedures
Low body mass index	
Severe kidney failure (III-IV stage KDOQI)	
Acute or decompensated hepatopathy	
Hypertension/heart failure (secondary to kidney disease)	
Untreated or undertreated hypothyroidism	
Diabetes mellitus	
Acute infection	
Biliary obstruction	
Major trauma with increased metabolic demand	
Vitamin D deficiency	
Genetic polymorphisms - Cytochrome P isoenzymes - Lipin-1 mutations - ABC transporter polymorphism - RYR gene variant - SLCO1B1 gene variant	

Abbreviations: ABC = ATP-binding cassette, CK = creatine kinase; KDOQI = kidney disease outcome quality initiative, RYR = ryanodine receptor, SAMS = statin-associated muscle symptoms, SLCO1B1 = solute carrier organic anion transporter family member 1B1, ULN = upper limits of normal.

## Data Availability

Not applicable.
